# Some Functions of the Dental Society

**Published:** 1913-11-15

**Authors:** N. S. Hoff


### SOME FUNCTIONS OF THE DENTAL SOCIETY.

BY N. S. HOFF, D.D.S.

Read before the Holmes Dental Club of Grand Rapids, October 31, 1913.

I was invited by your Chairman to discuss with you some functions of the dental society which might create a new interest and more enthusiasm for the work. To advance a new thought in this cause to-day, would require a keener imagination than I possess. If I could review the history of the dental society with enough detail to cite its career of failure and success, I could probably bring you some suggestions worthy of your careful consideration. For lack of time to do this, I will only cite by way of a general premise the fundamental facts, and then make a few observations that have come under my notice from time to time.

I think we may say in a few words, that the dental society is an opportunity to cultivate a sympathetic interest in the common work in which we find ourselves engaged.

I often wonder if some of the men in our profession know why they are in it. Most of us got into it by a mere accident, and as we have never had a real good chance to escape, we remain, and since we cannot get out, we try to make the most of it by constantly endeavoring to make ourselves more skillful and efficient servants. For the most part, we are honest, and are conscientiously endeavoring by our personal conduct and attainments to create a professional calling which shall, by ministering intelligently to human needs, entitle us to the commendation of our fellows.

In the early days of our calling, it was not a profession although a most useful avocation. It was practiced more as the art of the craftsman, than as the science and art of the scholar. But as the sphere of our usefulness enlarged, men were forced to take a larger and more humanitarian view of our work.

Formerly the dentist was to a large extent a charlatan; that is, he was selfish and narrow minded, even though tolerably well educated. The custom was to secrete knowledge, or at least to hord it for barter. A hundred years ago, there was no free discussion of ways and means in dental societies, and no printed books or dental journals willing and anxious to print every new discovery. But the time came when big men saw the beneficense of dentistry, and gave their lives to it. They were not content to hide their knowledge, they saw that the world needed it, and so they organized the dental society as a means of communicating to each other the ideas that came to them, and to discuss with each other their difficulties that they might get help in solving their problems. The dental society was born of necessity and it has continued to this day for the same reason, and there does not appear to be any other organization in view that will supplant it, at least not in the near future. It has become one of our most cherished institutions, and we could possibly better spare our colleges than our dental societies. Without it, we should make slow progress in proffessional development; our literature would suffer most severely; fraternally we should be in a hopeless condition indeed. The dental society is in fact the visible manifestation, not only of what we are, but also of what we are hoping and striving to become. It has taken possession of us so gradually and unconsciously, that we could not now abolish it and maintain our position as a learned proffession without a complete reorganization that would mean a practical revolution.

In its general form it has changed but slightly in all the years of its existence. We have improved it in some particulars. It is more democratic than when first organized. We still read papers and discuss them; but we are making more of the clinical features, and exhibits of various kinds, than was customary in the original society. We perhaps give more time to the consideration of purely proffessional matters, mainly however because there are greater demands and occasions now for this form of activity than in the beginning.

It has been charged that the dental society was being corrupted and exploited for selfish interests and personal aggrandizement. That men were using it to parade themselves and their superior knowledge or technical attainments before the public; that it was being used for promoting political cliques of office holders; that the dental supply houses were working it in their own interests to promote the business of selling supplies and obtaining material for publication in the journals published by them; also that it is a useless waste of the dentist's time and money which could be used to better advantage in endowing scholarships or research positions in the dental colleges, where better results could be had and with less effort. The latest criticism we have noticed seems to be directed at the present idea of the largely attended meeting. The criticism is that a mania for bigness has taken possession of our program committees, and they are striving to enlist the largest list of clinicians or eminent essayists, that a large crowd of observers may be attracted. The most objectionable feature being the methods used to advertise and promote the affair, some of them differing not at all from the flagrant announcement of the circus poster, or the medical nostrum vendors. An extravagant promise of a spectacular show that will attract a large crowd becaues of the novelty of the meeting and the general conviviality promised.

That each and all of these criticisms may be justified and are well founded in particular instances, can not be questioned. After all, can any really thoughtful and observant attendant on the sessions of the average dental society of the past, or present, admit that these degenerating influences have been strong enough to overshadow or nullify the good proffessional work accomplished by the society? Similar criticisms have been made of the work of almost every other useful organization we have, extending all along the line from the ward caucus to the church and legislative assemblies, but has any one ever said that we should withdraw our support from these causes because mistakes in management have occurred, or because unworthy individuals have exploited them for selfish purposes? The salvation army and the Red Cross Society have met similar criticisms, but they have withstood them and are stronger in public favor today than ever before, and are accomplishing untold good in the world.

Because some discontented or garrulous knocker thinks the dental society unworthy of his support, he can make enough noise to alarm the entire profession and do incalculable harm to the best institution we possess for real professional promotion. From the very nature of the case we must expect our dental societies to make mistakes and fall below our ideals of perfection in organization and efficiency of function. If we could command a supreme wisdom and a disinterested executive control, we might expect entire satisfaction and a more cordial co-operation of the profession. Since we are all very human, we should not demand perfection even though we believe it is due us. Sometimes the best way to cure the chronic knocker is to put him to the test by placing him in the responsible position. He and all of us will ultimately come to see that to attain ideal results we shall have to have ideal co-operation. The things we want to see done in our societies that seems to us of supreme importance we shall have to help put there by cordial and helpful co-operation. It is our right to criticize but this implies also a moral obligation that becomes a duty, and we are forced to put our best energies into the work that we may exert an influence that is coercive, and will lead to the consummation of our high ideals. It will not answer for us to dissent, or worse yet, desert the good ship and let her go down, while we stand off and criticize those who are struggling with the pumps as best they can. If the dental society of today is not what our present enlightenment demands, it certainly is our privilege and duty to bring its work up to our standards of ethical and professional attainments. It is wrong for us to say we cannot do this because the politicians have control and we can do nothing. A political clique can not long stand out against a righteous sentiment. If we know our work is faulty let us correct it and not waste time or energy by submitting to selfish interests or inefficient management.

It is not probable that any better organization will supplant the dental society in the near or distant future, it is our most cherished heritage, and we can not escape the responsibility of keeping it free from abuse or destruction. If we are wise, we shall act quickly and insistently to save it intact.

As members of the Holmes Dental Club, what are we here for this evening? What is there that we should do that will challenge the best that is in us and so compel our enthusiastic co-operation? Are we in the critical or fault finding state of mind, or are we indifferent? Is there not some idea that you want developed, or some troublesome item of experience or practice that you need more light on, which you can trust your confreres here to put you right about? Of course your essayist does not know you individually, and can have no adequate conception of your personal or professional problems. I am compelled therefore to generalize my statements, with the hope that some suggestion may cause a discussion that will be more pointed and practical than anything I shall be able to present to you. If this should transpire, I shall have accomplished that for which I have come.

The first and most important suggestion that I can make to you is that you determine among yourselves what it is that you need most to know or do. There surely is something that you believe you should know or do at once if practicable. Then I would suggest that you undertake this problem in the most earnest and intelligent manner that is possible with your organization. In short, find something that appears to you as worth while doing, and then tackle it with all your heart and strength.

I am aware that this advice contains nothing remarkably new and novel, and it may not appeal to you because you say you are not out for trouble. Or you may say there are so many things we want to know and there are so many different notions about what is the best thing to do, and above all, we don't know how to tackle such problems because we are not working along those lines. We shall have to send to Ann Arbor or Detroit, or Chicago, to get an expert to come over and show us how to do those things.

Now I will explain why I made the above statement. I said you should agree to start something, not that I expect a unanimous agreement on anything—that is probably impracticable—as some member will surely object and want something else or some other method adopted. It is just as well that you meet some opposition, but the majority, or the concensus of opinion will enable you to come to a decision, and the struggle, if honorably conducted, will be wholesome if all abide by and accept its conclusions.

After you have decided what you want done, the next best thing is to decide how it shall be done and who shall undertake it. Here again you may make mistakes and there is a big chance for failure; because you may decide on a bad method, or you may make a bad selection for a leader, or of his assistant helpers. The majority again rules, and the most capable and aggressive leader should be put in charge, even though some one's feelings are hurt and the harmony of the organization is threatened. It may be that the end of the experiment will be the entire disruption of the society.

I cite this plan because it is typical of what has happened in many other societies. Disaster comes in one form or another in so many cases, that we are inclined to doubt its practicability, and yet it is so natural and sensible a method that we wonder why it ever fails. The secret is that we find in every organization the elements of inharmony, which develops inordinately when there is a chance for difference of opinion. The moral is that no successful work can be expected in any organization where there is a persistent element of inharmony. *We must get together. We must stand and work together.* If this is impractical there is not much hope that you will accomplish your desires, or anything else worth while.

I want to say here also that the work of an organization like yours can be crippled if not wholly killed, by too much legislation and machinery. Don't do too much work in the form of resolutions and motions and reference to committees. This is a favorite method of narcotizing well meant schemes. Resolutions are recorded and forgotten. Committees neglect their work, or report progress until they finally ask to be discharged because they become ashamed of their inactivity. Many times a case in practice will start a discussion that will interest and be informing to the entire meeting; when the report on the case by a special committee to which it has been referred, will elicit little or no debate. Some things will need to be referred, in order that the time of the society may not be consumed by a fruitless discussion, or that fuller information may be forth coming, but a general and informal discussion will usually be more interesting and instructive than a formal and well considered one. in which the general body has no part.

It will be better usually to have the program outlined, in order that something of value will be forth-coming at each session, and we do not wish to be understood as endeavoring to discredit well considered and formal papers and exhibits. These are invaluable in their proper place, but they should not monopolize the entire meeting, as this plan contracts unduly the opportunity for general participation.

If a formal program is designed and specific work is given out, great care should be exercised to select capable and interested men on the committees, having charge of such work. Select a man whose habit it is to do things and who knows how not only to do the thing, but who will associate with himself, two or three other men who are willing to work with him and who will themselves be benefited by doing some good work on the committee. It will increase their loyalty and at the same time put them in training for future work. A small committee under a capable leader will do better work than a large committee, which is likely to neglect or shirk its assignment. Sometimes these committees can be so constituted as to bring together socially men who ought to know each other better, and warm personal life friendships are made with great benefit to the profession and society work. Some of my most valued friends, I met the first time in this way, and I have gotten many an inspiration to work for the good of my profession from these men. In truth, I can say that these intimate and personal touches that I have had with the great men of our profession have not only been a great and continuing pleasure, but they have kept me steadily on the alert to be a better professional man, and to use my strength wholly for professional betterment. On the other hand one of my greatest pleasures has been to start young men on the right track for a successful career in their calling. This fact ought to be sufficient incentive to urge the older and more experienced men in your society to act as leaders of at least one or two younger men who will be glad to undertake some work which they can do for their profession through the local, state, or national society, if they can have the help and encouragement of some one older and more experienced man to lead or encourage them.

If things are going slow in your society, and there is a lack of interest there is a reason for it. Your plans are not well developed or the society does not sympathize. A one man plan needs lots of steam to push it along, but a united body behind a poor plan will accomplish something without great force or genius. A special effort should be made to interest in some active work the young men and the quiet or reserved older men. The men who warm the chairs do a lot of thinking, and they often have better ideas than are expressed on the floor. A real star may be occupying one of your benches, whilst you are going from bad to worse, for lack of his assistance. Try out these men in a favorable way and you will get a spark where you least expect it sometimes. There are lots of stored up dry cells that only need connecting up to get the energy.

As to materials for consideration, I have only to suggest that you undertake live subjects, don't waste your energies considering thread bare topics, neither is there anything to be gained by giving too much time to fads that depend upon impracticable conditions for the most part to make them serviceable. There are plenty of live topics that need to be discussed from the clinical standpoint, and an experience meeting occasionally on how you are succeeding with some material, or method of practice that is occupying the professional mind at the time, will get out some of the quiet people and if they don't take part in the open discussions, they will confer with each other after the meeting, or during the social hour.

It will be a good thing to keep in mind the fact that every member who takes part in a meeting will feel repaid for the trouble and time spent in attending the meeting. These are the men who will make the remark that it was a good meeting. Therefore select interesting topics and get as many as possible to contribute a few words to the discussion or present a case in practice, as illustrative. This places a responsibility on each member and commits him to the work of the society.

A spirit of candor and freedom without overbearing or insinuating censure should prevail. If anyone has a grouch, rule him out of order, and set aside a committee meeting or a special opportunity for a complete hearing and adjustment of his case. Nothing is to be gained by harsh criticisms, and many a modest but capable young man has lost interest and enthusiasm for society work because he has been unkindly censured or has seen his freinds unfairly dealt with. Hardly two dentists can be found who will even think alike, much less do a piece of technical work in the same way. We should permit the greatest latitude and freedom of speech with kindness and consideration for the opinions and ideas of others.

Don't permit the formation of a clique of politicians, or social interests to even seemingly have the management or control of your society. We should rise above such things and meet as a fraternity in which all are honored alike. Preferment should be given only to him who serves best. It is fine to worship a hero, but degrading to have to serve a boss.

In our earnest desires to equip ourselves with new technical ideas or better knowledge of the scientific principles involved in the practical details, we generally fill our programs with technical and scientific subjects to the exclusion of other things that might contribute to the interest of our proceedings. It is true we have introduced the banquet with its smokes and social intercourse, all of which is good because it helps to get us better acquainted with each other and makes for good comradship. I have often wondered why it would not be a good plan to have a ladies' night when we could gather about the table with our wives and sweethearts and afterwards have a program that would give our wives some idea of what we do at these meetings. I believe we would have better papers if the contributors knew their wives were going to be present to hear them and I am not sure but our wives would not only be more lenient with us when we are spending the time they think should be theirs in preparing for the society meeting. I believe also that they would spur us on to do our best, as they are proud of us, and they don't want us to shirk our duty or do an inferior bit of work. It is a common remark of successful men that they never could have done it except for the help of their wives. Have we not made the mistake of keeping our professional life with its cares and disappointments away from our wives and so lost the inspiration and help that comes from the one who will give us the most needed and unbiased advice? How can we expect our wives to help us, or rather why should we expect them to be even interested in our professional advancement, when they hav'nt the slightest idea of what the professional life means to us, or what we ought to be doing other than to serve the patients who come to our offices as faithfully as we can, for business reasons only. How many here present have ever talked over your professional ambitions with your wives? Can we blame them if they complain when we take a night off for the local society, or a week away from home for the state or national meeting? If we do not confide to them our ideals, we should not expect their sympathy or co-operation. It seems to me there is an opportunity here to do a little home missionary work that has been sadly neglected, which will yield ample results in cultivating better social and professional relations, and which may be the means of cementing mere friendships or speaking acquaintanceships into genuine fraternal relationships. At least a professional comradery would be developed that will be more comfortable and helpful than our present condition of social segregation can ever be. I do not doubt that the influence of some such a movement would react upon the members of the profession in any community where this matter was introduced in the right way to the distinct advantage of the profession. The social life would be more comfortable, the professional relations would be more ethical, and there would be a greater striving for professional excellence, with a distinct advantage resulting to the people of that community, because of the higher order of service administered to them.

I don't know how many meetings you hold each year, but I make an earnest plea for at least one ladies' night and next summer a picnic for social purposes only.

As an illustration of what can be done to promote professional good fellowship, I would cite the case of one of our cities which has a dental population of about 35 practitioners. Ten years ago, there were three rank advertisers in that town, and a decent fee for a high-grade service was thought impossible. A dental club was organized at first to buy dental supplies at wholesale. It has now grown into a social dental society and the supply purchasing function is the least important of its considerations. Practically every dentist in the city is a member of the organization. All the advertisers have left the city with a single exception, and he is a very moderate offender. Two young men located there five years ago, expecting to start an advertising office. They were promptly taken into the dental society and shown the better way, both socially and professionally, and they are to-day practicing ethically and are very active workers in the society. Every dentist in that city is as busy as he wants to be, and the average fees for service are better than in any city of its size in the state that I know of. The social and fraternal side of the profession has been carefully looked after in a moderate and sensible way, and at the same time there has been no neglect of the professional ideals.

Now I don't know that I have brought you any new idea or suggestion that you can cast into concrete form for future action. I hope, though, to have opened up the subject for a good discussion that will result in extending the activities of your society in such directions as may increase your loyalty to the profession, and stimulate your love for its artistic and scientific development, and that you may have your best year's *work* before you.

In closing I want to make one more suggestion that I feel is of the greatest importance. You all know about the scheme for reorganizing our society activities so that the local and district societies become parts of and co-workers with our state and national societies. I believe this to be one of the greatest advances ever made by our profession; and the benefits that will come to us will be measured in the exact proportions that our several organizations commit themselves to the scheme. Many objections have been made to the plan and some men and societies are still withholding their affiliation. But enough have signed up to assure the success of the venture, and it will materialize at an early date. What a grand thing it will be to have the entire profession of the United States working together with one idea in view. What a tremendous impulse this will give to our professional development. When all the plans are matured, we shall have before us the printed transactions of all the important societies in the country, and at a nominal cost. It seems too good to be true, but we are assured that it will surely be carried out if the profession will do its part. What I want to suggest is that every member of this society makes up, before January 1st, his dues to the district, state, and national societies, so that our state society can place an undivided society immediately in the National Association. I am so confident of the great benefits that shall ultimately come to us from this movement, that I cannot understand why anyone should hesitate for one minute to give his cordial support to the movement. I hope your society has, or will do this thing, as I am sure you will receive much more than you give, and the influence of your action will have a salutary effect upon other organizations.

